# Interactions Between Nutraceuticals and α-Synuclein Conformational States: Molecular Mechanisms and Neuroprotective Implications in Parkinson’s Disease

**DOI:** 10.3390/ijms27031324

**Published:** 2026-01-28

**Authors:** Bruna Amenta, Rosalba Minervini, Maria Laura Matrella, Tiziana Cocco

**Affiliations:** Department of Translational Biomedicine and Neuroscience “DiBraiN”, University of Bari ‘Aldo Moro’, 70124 Bari, Italy; bruna.amenta@uniba.it (B.A.); r.minervini11@studenti.uniba.it (R.M.)

**Keywords:** α-synuclein, protein misfolding, conformational landscape, nutraceuticals, neurodegeneration

## Abstract

Synucleinopathies, including Parkinson’s disease (PD), are neurodegenerative disorders characterized by aberrant aggregation of α-synuclein (α-syn), a presynaptic protein with an intrinsic disorder nature. The transition of soluble monomers into oligomeric and fibrillar species represents a key molecular event driving neuronal dysfunction and neurodegeneration. Emerging evidence suggests that nutraceuticals, bioactive compounds derived from dietary sources, can modulate α-syn aggregation at multiple conformational stages. Polyphenols, alkaloids, ginsenosides, and food-derived peptides interfere with α-syn structure and assembly, suppressing the formation of toxic oligomer species and promoting the clearance of misfolded assemblies. Despite this potential, clinical translational of nutraceuticals is currently limited by poor systemic bioavailability and restricted central nervous system penetration due to blood–brain barrier constraints, which have largely confined research to preclinical studies. In this context, this review summarizes current knowledge of nutraceutical interventions targeting the conformational landscape of α-syn and highlighting both direct and indirect molecular mechanisms with involved in aggregation-prone species. Furthermore, we critically examine key challenges related to bioavailability and clinical translation, focusing on advanced delivery systems and precision-based approaches to enhance neuroprotective efficacy and support the potential of nutraceuticals as novel or adjunctive therapeutic strategies for PD.

## 1. Introduction

Parkinson’s disease (PD) is the most common form of α-synucleinopathy, a group of degenerative disorders characterized by the pathological misfolding and accumulation of the presynaptic protein α-synuclein (α-syn). This family also includes dementia with Lewy bodies (DLB) and multiple system atrophy (MSA). In DLB, α-syn deposits occur in neurons [1], while in MSA, they appear in oligodendrocytes [2,3]. PD is clinically defined by a combination of motor and non-motor features that reflect its multisystem nature [4]. The progressive loss of dopaminergic neurons in the substantia nigra results in classic motor symptoms, including tremor, bradykinesia, rigidity, and postural instability. However, symptoms such as sleep disturbances, autonomic dysfunction, and cognitive decline are now recognized as integral components of the disease, indicating widespread involvement of neurotransmitter systems beyond the nigrostriatal pathway [5,6]. DLB is characterized by early cognitive impairment, fluctuating attention, and recurrent visual hallucinations, reflecting the widespread cortical and limbic distribution of α-syn pathology [7].

In contrast, MSA is defined by glial cytoplasmic inclusions, rapid clinical progression, and limited therapeutic response, combining Parkinsonism with autonomic and cerebellar dysfunction [8]. Initially identified as a neuron-specific protein localized to nuclei and presynaptic terminals [9], α-syn gained prominence when Spillantini et al. demonstrated its presence in Lewy bodies, establishing a direct link between α-syn accumulation and neurodegeneration [10]. Before this discovery, Lewy bodies were primarily identified by their strong ubiquitin immunoreactivity, which was considered the most reliable marker at the time [11]. Subsequent studies revealed that Lewy body filaments consist predominantly of full-length α-syn and exhibit stronger immunoreactivity than ubiquitin, making α-syn staining the gold standard for detecting Lewy pathology and redefining the neuropathological framework of the synucleinopathies [12]. α-syn is a small protein encoded by the SNCA gene, abundant in the brain and specifically enriched in presynaptic terminals of neurons [9]. At the molecular level, α-syn exists as a dynamic ensemble of conformations that facilitate transitions between soluble monomeric states, physiological multimeric assemblies, and pathological aggregated species, unable to determine cellular dysfunction [13]. In Lewy pathology, α-syn frequently co-aggregates with neurofilament proteins, linking cytoskeletal disruption to neurodegeneration and highlighting its dual role as both a pathological hallmark and a mechanistic driver of disease [14]. The intrinsic conformational plasticity is further modulated by the post-translational modifications, among which phosphorylation of Ser129 is the most prevalent. Detected in most pathological aggregates, Ser129 phosphorylation has consistently been associated with an increased propensity of α-syn to aggregate [15].

Beyond its pathogenic relevance, this modification also carries diagnostic significance, while α-syn itself is emerging as a measurable biomarker in cerebrospinal fluid (CSF) and blood for the diagnosis of α-synucleinopathies [16,17,18]. Physiologically, α-syn contributes to synaptic homeostasis by regulating synaptic vesicle dynamics, promoting the assembly of soluble N-ethylmaleimide-sensitive factor attachment protein receptor (SNARE) complex and facilitating neurotransmitter release, exocytosis, endocytosis, and vesicle recycling [19,20]. However, under stressful conditions or pathological overexpression, the α-syn protein becomes unstable, which destabilizes its conformational landscape and promotes misfolding and aggregation. This process is consistent with evidence that implicates synucleins in both synaptic function and neurodegeneration [21,22,23]. Protein misfolding and aggregation represent the pivotal transition from physiological to pathological states, with the central non-amyloid-β component (NAC) domain playing a critical role in fibril formation [24], though a multistep process involving nucleation, elongation, fibril maturation and prion-like propagation [25,26]. The high level of heterogeneity makes it difficult to isolate and study the toxic properties of the different co-existing aggregate species and determine which fraction is toxic [25,27,28]. In this context, accumulating evidence suggests that soluble prefibrillar and oligomeric α-syn species, rather than mature fibrils, are the primary mediators of cellular toxicity in PD. Multiple studies demonstrate that small, non-fibrillar α-syn aggregates lead to membrane disruption, abnormal calcium influx, neuroinflammation responses, and neuronal dysfunction, whereas monomeric and mature fibrillar assemblies exhibit comparatively limited acute toxicity [25,27,29]. At the mechanistic level, oligomeric α-syn species exhibit distinct structural features, including exposed hydrophobic surfaces, increased β-sheet content, and pronounced conformational flexibility, which promote aberrant interactions with cellular membranes and organelles. These interactions lead to mitochondrial dysfunction, endoplasmic reticulum stress, synaptic impairment, and proteostasis failure, properties that are largely absent in mature fibrils [30,31,32]. From a biophysical perspective, oligomers represent metastable and structurally plastic intermediates that trigger the initial collapse of neuronal homeostasis, whereas fibrils correspond to a more stable downstream aggregation state and may act as relatively inert end-stage deposits [29,33]. Consistently, in vivo studies show that α-syn variants favoring oligomer formation induce greater dopaminergic neurodegeneration than rapidly fibrillizing species, establishing conformational intermediates as the most disease-relevant and therapeutically actionable aggregation states [25].

**Figure 1:**
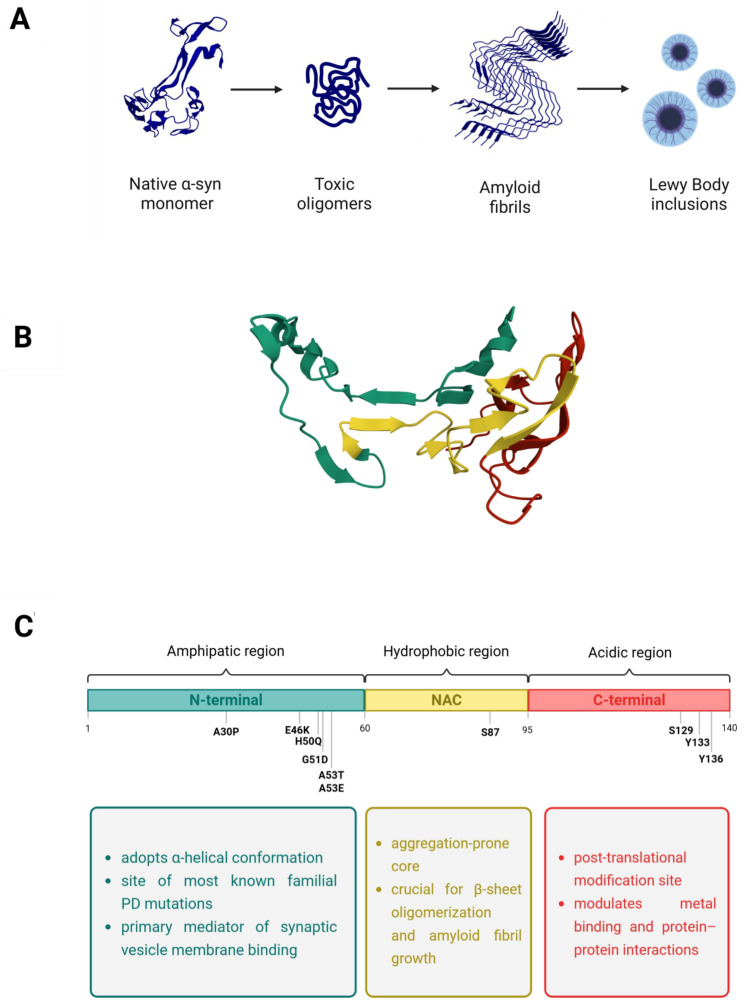
Structural and domain organization of human α-syn. (**A**) Schematic representation of α-syn aggregation pathways. Native α-syn monomers undergo misfolding to form soluble oligomeric species, which subsequently assemble into insoluble β-sheet-rich α-syn fibrils. These amyloid fibrils further accumulate into Lewy bodies, the pathological hallmark inclusions of PD and related synucleinopathies. (**B**) Representative conformer of human α-syn rendered from the RCSB Protein Data Bank (PDB ID: 9A1A). The N-terminal amphipathic region (aa 1–60), the central non-amyloid-β component (NAC, aa 61–95) and the C-terminal acidic tail (aa 96–140) are color-coded in green, yellow and red, respectively. α-helices and β-strands are shown as ribbons and arrows, respectively. (**C**) Linear schematic of the α-syn sequence, subdivided into N-terminal, NAC and C-terminal regions using the same color code. Vertical arrows indicate familial PD-associated mutations in the N-terminal domain (A30P, E46K, H50Q, G51D, A53T, A53E), as well as key regulatory residues in the NAC (S87) and C-terminal (S129, Y133, Y136) regions. Functional roles and pathological relevance of each domain are summarized in the color-matched boxes below.

Within an energy-landscape framework, the intrinsically disordered nature of α-syn enables a dynamic equilibrium among monomeric, oligomeric, and fibrillar conformers, a process finely modulated by disease-associated mutations, post-translational modifications, and environmental factors [34,35] (Figure 1A). This conformational plasticity has important implications for therapeutic targeting.

Small-molecule drugs are low-molecular-weight compounds derived from synthetic or natural sources that typically exhibit stable structures, enabling efficient membrane permeability and selective interactions with biological targets, including enzymes, receptors, and proteins [36]. However, conventional structure-based small-molecule drug design is limited in the context of α-syn by the lack of a stable tertiary structure and well-defined binding sites [37,38,39]. Consequently, interactions between small molecules and α-syn generally induce minor shifts within its conformational ensemble. Unlike structured proteins, these interactions do not lock α-syn into a fixed inactive state but instead remain weak, transient, and highly dynamic [37,40].

In this context, nutraceuticals have emerged as promising modulators of α-syn aggregation and toxicity. Increasing evidence indicates that natural compounds, such as flavonoids, polyphenols and other phenolic derivatives, can interact with the different conformational states of α-syn, thereby preventing oligomer formation, destabilizing fibrils and promoting the conversion into less toxic assemblies. Specifically, flavonoids have been shown to remodel α-syn fibrils into amorphous aggregates with a lower seeding capacity [41,42,43], whereas polyphenolic compounds such as (−)-epigallocatechin gallate EGCG can break up preformed oligomers [44,45]. Furthermore, other phenolic molecules inhibit oligomerization and attenuate α-syn-induced synaptic toxicity [46]. Recent systematic reviews focused on natural products that interfere with α-syn aggregation or promote its clearance, highlighting their potential role as disease-modifying agents and therapeutic interventions for PD [47,48]. Integrative approaches further emphasize nutraceuticals as a conceptual bridge between nutrition and neuroprotection [49], offering complementary strategies to attenuate PD progression [50,51]. While previous reviews have broadly explored the neuroprotective potential of natural compounds in PD, mainly focusing on antioxidant, anti-inflammatory, and metabolic pathways, the present review adopts a distinct mechanistic perspective. Specifically, it considers α-syn as a dynamic intrinsically disordered protein and examines how nutraceuticals modulate its conformational ensemble and aggregation pathways, thereby linking these effects to protein homeostasis and neuroprotection. to protein homeostasis and neuroprotection.

## 2. α-Synuclein: Structure and Conformational Dynamics

α-syn is a small acidic intrinsically disordered protein (IDP) of 140 amino acids (~14 kDa) mainly present at presynaptic sites, where it regulates synaptic vesicle dynamics, neurotransmitter release and synaptic maintenance [52,53,54]. These functions are mediated by its ability to interact dynamically with lipid membranes and components of the SNARE complex, supporting synaptic maintenance and plasticity [53]. However, its structural plasticity that enables physiological function also confers an intrinsic vulnerability to misfolding into toxic, insoluble fibrils, such as those found in Lewy bodies, that characterized the α-synucleinopathies [30,55]. Structurally, α-syn is organized into three functional regions that determine its conformational behavior and aggregation propensity (Figure 1B) [56]. Specifically, the N-terminal region (residues 1–60) contains a series of amphipathic repeats that form an α-helix structure, crucial for its membrane binding capability (Figure 1C) [57]. The central non-amyloid-β component (NAC) domain (residues 61–95) is a hydrophobic region and constitutes the core aggregation-prone region, driving β-sheet formation and fibril assembly (Figure 1C) [58]. Finally, the C-terminal acidic domain (residues 96–140) mediates interactions with calcium ions, molecular chaperones, and synaptic proteins (Figure 1C) [59,60]. Although α-syn was initially described as a natively unfolded monomer, subsequent evidence has shown that the endogenous protein can physiologically assemble into folded tetramers of approximately 58 kDa, which exhibit minimal propensity for amyloid-like aggregation [61]. However, the existence and physiological relevance of tetrameric α-syn remain debated, with alternative interpretations proposing that multimeric species may result from dynamic, context-dependent assemblies rather than representing stable, native conformations [62]. Together, these findings revealed the existence of a dynamic equilibrium between monomeric and multimeric α-syn species, which enables transitions between disordered and aggregation-prone states [62]. This conformational landscape may support physiological function while simultaneously conferring vulnerability to misfolding and aggregation under pathological conditions [63]. At the monomeric level, α-syn behaves as an IDP, lacking a stable tertiary structure and sampling a heterogeneous ensemble of conformations [64]. Consistently, single-molecule force spectroscopy (SMFS) studies have revealed that α-syn can adopt both extended and compact conformations, which interconvert slowly and are strongly influenced by environmental conditions [65,66]. In this context, lipid interactions play a central stabilizing role [67]. Specifically, in the presence of membranes, the N-terminal region adopts α-helical conformations that stabilize association with synaptic vesicles [68]. Conversely, membrane detachment or alteration in lipid composition increases exposure of aggregation-prone segments, particularly in the NAC domain, facilitating the transition towards β-sheet-rich conformations and promoting aggregation [69]. Cryo-electron microscopy (cryo-EM) has provided the first atomic models of α-syn fibrils, revealing helical filaments composed of β-sheet-rich protofilaments [70]. These structures explain the seeding capacity of fibrils and their toxic impact on cellular homeostasis [71]. The cellular environment further modulates this conformational landscape. Notably, phospholipids promote fibril elongation, whereas oxidative modifications facilitate the conversion of soluble monomers into aggregation-prone protofibrillar species [72,73]. In addition, genetic mutations linked to familial PD, including A30P, E46K and A53T, also promote α-syn nucleation and fibril amplification, leading to earlier disease onset [74,75]. Conversely, small-molecule modulators, including osmolytes, low molecular weight organic molecules, polyphenols, peptides, and synthetic compounds, can interfere with α-syn aggregation by targeting distinct stages of the amyloidogenic pathway. More specifically, these molecules may promote the formation of native or compact conformations, disassemble preformed aggregates, or modulate cellular proteostasis mechanisms indirectly [76,77,78]. Advanced techniques, including nuclear magnetic resonance (NMR), cryo-EM, and SMFS, have deepened our understanding of these structural transitions, underscoring the therapeutic value of targeting early aggregation intermediates [79]. In this context, NMR and SMFS are particularly useful for investigating modulator interactions with monomeric and early oligomeric α-syn [66,80,81]. In contrast, cryo-EM mainly provides structural information about the effects of ligands on stable oligomeric or fibrillar assemblies [82,83]. A recent integrative approach combining SMFS and native mass spectrometry has revealed that α-syn monomers exist as ensembles of random coil and compact states, with ligands such as dopamine and EGCG shifting the equilibrium toward compact, less aggregation-prone conformers [66,84]. Using cryo-EM, Chen and his co-authors described the structure of stable oligomers of α-syn that have emerged as key contributors to toxicity by impairing membrane integrity and facilitating further aggregation of the protein [85]. Collectively, these findings suggest that α-synuclein can adopt multiple oligomeric and fibrillar assemblies, reflecting distinct pathological strains which have been linked to the clinical and molecular heterogeneity of synucleinopathies [55,86]. Consequently, α-synuclein aggregates do not represent a uniform molecular target, and interactions with modulators, including nutraceuticals, are likely to depend on the specific conformational state or aggregate surface involved.

## 3. Mechanisms of Action of Nutraceuticals on α-Synuclein Misfolding

**Figure 2:**
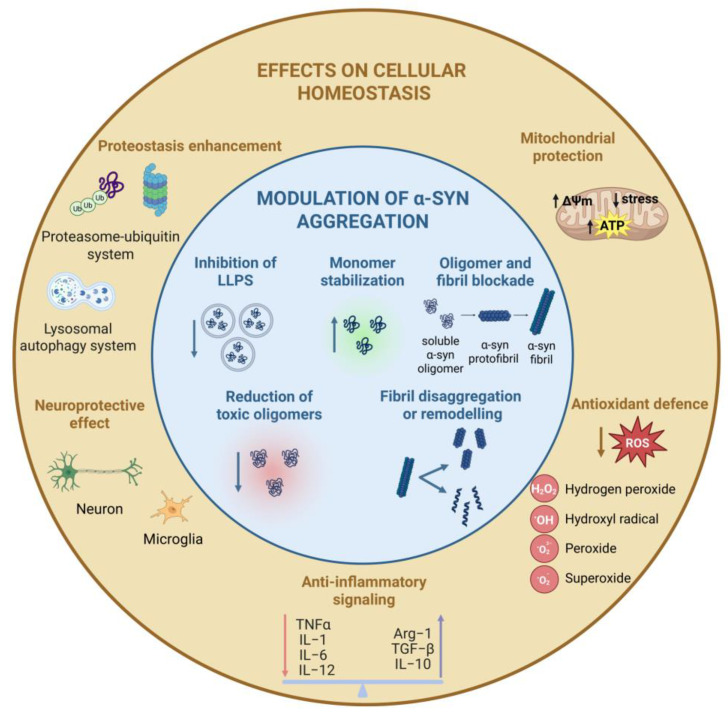
Mechanisms of action of nutraceuticals against α-syn pathology. Nutraceuticals counteract α-syn-associated pathology through convergent actions on the aggregation cascade and its downstream consequences. The inner blue circle summarizes anti-aggregation mechanisms, including inhibition of liquid-liquid phase separation (LLPS), stabilization of monomeric and non-toxic conformations, prevention of oligomer and fibril formation, reduction in or destabilization of toxic oligomeric species, and fibril disaggregation or remodeling to reduce aggregate-associated toxicity. The outer brown circle highlights homeostasis-restoring pathways that may contribute to disease progression, involving enhancement of proteostasis (chaperone activity, correct folding, and clearance through the ubiquitin-proteasome and lysosomal-autophagy systems), improvement of antioxidant defenses (scavenging reactive species and boosting endogenous systems to limit oxidative damage to lipids, proteins, and nucleic acids), preservation of mitochondrial function (manteinance ΔΨm, improved ETC efficiency, and sustained ATP production), attenuation of pro-inflammatory signaling, and overall neuroprotective effects.

Nutraceuticals are bioactive compounds derived from natural food sources with therapeutic properties. Their discovery has led to the development of alternative strategies for addressing neurodegenerative diseases. At the molecular level, small bioactive compounds can interact with α-syn monomers, oligomers, or fibrils, modulating the kinetics of primary nucleation, elongation, and especially secondary nucleation on fibril surfaces, which represents the main source of toxic oligomers under near-physiological conditions [87]. Nutraceuticals can modulate abnormal aggregation of a-syn through two complementary mechanisms. First, they can directly interfere with the aggregation process, preventing liquid-liquid phase separation (LLPS), blocking formation of oligomers/fibrils, stabilizing monomeric or non-toxic conformations (as shown for curcumin, olive polyphenols, etc.) [46,88,89,90,91]. Second, nutraceuticals can act by destabilizing/disassembling existing aggregates or redirecting aggregation into non-toxic species through disaggregation of fibrils, remodeling of oligomers/fibrils into less toxic forms, prevention of toxic oligomer formation, or aggregate-toxicity reduction (Figure 2) [41,44,92,93,94,95,96].

Nutraceuticals may also attenuate disease progression indirectly by modulating key cellular pathways that maintain physiological homeostasis, including proteostasis, redox balance, mitochondrial function, and neuroinflammation [50,97,98,99,100,101]. Specifically, these compounds influence processes that are compromised in many neurodegenerative conditions, such as proteostasis by enhancing chaperone activity and promoting the clearance of misfolded or aggregated proteins, thereby supporting the correct protein folding (Figure 2) [102,103]. Moreover, through direct scavenging of reactive oxygen and nitrogen species, as well as by upregulating endogenous antioxidant defenses, these bioactive compounds limit oxidative damage to lipids, proteins, and nucleic acids (Figure 2). Many nutraceuticals, including polyphenols, carotenoids, vitamins, and bioactive peptides, also exhibit mitochondrial-protective effects [104,105,106], by preserving mitochondrial membrane potential, improving electron transport chain efficiency, reducing mitochondrial reactive oxygen species (ROS) generation, and supporting optimal adenosine-5′-triphosphate (ATP) production (Figure 2) [107,108,109]. In addition, many nutraceuticals exhibit anti-inflammatory properties, attenuating neuroinflammatory signaling pathways that contribute to neuronal injury and disease progression (Figure 2) [110,111].

Overall, these combined effects support cellular resilience and underscore the potential of nutraceuticals as complementary strategies to conventional therapies [100,101]. Nutraceuticals can modulate the conformational landscape of α-syn by inducing specific structural rearrangements through distinct chemical scaffolds and interaction mechanisms [95,112]. They can be grouped into distinct families, each characterized by distinct mechanisms of action determined by their chemical structure and functional groups.

However, this significant structural diversity makes it difficult to identify a single common mechanism, as different chemical scaffolds may engage α-syn through different interaction pathways [44].

Plant-derived compounds investigated for their anti-aggregation and neuroprotective properties mainly belong to the polyphenol family, a large class of secondary metabolites involved in protection against ultraviolet radiation. This family of compounds have been widely studied for their health-promoting effects in cancer and chronic disease [113,114]. Within this group, flavonoids represent the largest and most structurally diverse group of polyphenolic compounds, including bioactive molecules such as baicalein, kaempferol, quercetin and EGCG [115]. Other relevant polyphenolic subclasses include stilbenes, such as resveratrol, which are synthetized by plants in response to microbial infection [116], as well as neoflavonoids, including brazilin, and curcuminoids, such as curcumin [117]. Within the polyphenol family, secoiridoids constitute a distinct group of monoterpenoid phenolics, with oleuropein being the best-known example, exhibiting potent antioxidant, anti-inflammatory, and anti-amyloidogenic activities [118].

Beyond polyphenols, additional nutraceutical families have also demonstrated neuroprotective potential. Triterpenoid saponins, particularly ginsenosides, such as ginsenoside Rb1, exhibit protective effects largely mediated by the preservation of mitochondrial homeostasis, activation of phosphatidylinositol 3-kinase(PI3K)/protein kinase B(Akt) and response element-binding protein(CREB)-brain-derived neurotrophic factor (BDNF) signaling pathways, and suppression of neuroinflammatory responses [119]. Alkaloids are also gaining attention for their neuromodulatory properties. Specifically, caffeine, a methylxanthine purine alkaloid, acts primarily as a non-selective antagonist of adenosine A_1_/A_2_A receptors [120], whereas nicotine, a nicotinic alkaloid, modulates neuronal activity through nicotinic acetylcholine receptors (nAChRs), notably α4β2 and α7 subtypes [121].

### 3.1. Polyphenols

Polyphenols represent a structurally diverse class of plant-derived metabolites, commonly found in fruits, vegetables, cereals and tea. Owing to their well-documented antioxidant, anti-inflammatory, and anti-amyloidogenic properties, these compounds have gained increasing relevance in neurodegeneration research [95,122,123]. Despite sharing a core aromatic ring with one or more hydroxyl groups, polyphenols include a wide range of subclasses, such as flavonoids (e.g., baicalein, kaempferol and quercetin), catechins (e.g., EGCG), stilbenes (e.g., resveratrol), secoiridoids (e.g., oleuropein), and curcuminoids (e.g., curcumin). Variations in their chemical structure, such as ring substitutions, degrees of conjugation and glycosylation patterns, can significantly affect their interactions with α-syn and their ability to modulate cellular pathways [124].

In this context, Caruana et al. conducted a systematic comparison of structurally distinct polyphenols demonstrating that specific molecular motifs strongly influence their ability to directly inhibit or disassemble α-syn oligomers [44]. Specifically, aromatic recognition elements and vicinal hydroxyl groups on a single phenyl ring were identified as key drivers of this direct mechanism of interaction, which typically involves the binding of polyphenol aromatic rings to the hydrophobic NAC region of α-syn, thereby preventing the adoption of β-sheet-rich pathogenic conformations [44]. In addition to these direct protein interactions, polyphenols can reduce α-syn thought indirect mechanisms, including the attenuation of oxidative stress and enhancement of autophagy-mediated clearance [100,125,126,127]. ROS are known to accelerate α-syn misfolding through post-translational modifications, such as tyrosine nitration and the formation of 4-hydroxy-2-nonenal (HNE) adducts, which promote the stabilization of toxic oligomeric species [128,129]. By limiting these redox-driven modifications, polyphenols may therefore indirectly counteract α-syn aggregation.

Taken together, these observations support the view that polyphenols act at multiple levels of the α-syn aggregation landscape, making them promising candidates for therapeutic intervention. The effects of selected polyphenols and the experimental methods used to evaluate their impact on α-syn conformational states are summarized in Table 1, while experimental models and concentration ranges are detailed in Supplementary Table S1.

#### 3.1.1. Baicalein

Baicalein is the main component of *Scutellaria baicalensis* and exhibits multiple biological activities, including antioxidant and anticancer properties driven by the modulation of several signaling pathways [130,131].

Bacalein has been shown to modulate the aggregation of different amyloidogenic proteins. Specifically, Nabi et al. reported that baicalein modulates the aggregation of amyloid-β42 (Aβ42), a hallmark of Alzheimer’s disease pathology, by both reducing fibril formation and promoting the disassembly of pre-formed fibrils [132]. At a mechanistic level, baicalein interacts with the aggregation-prone region of Aβ42, disrupting the Asp23-Lys28 salt bridge, a key stabilizing interaction required for β-sheet formation. This shifts the peptide toward α-helical conformations, thereby lowering β-sheet content and impairing amyloid assembly [132]. Baicalein has also been shown to modulate Tau aggregation, inhibiting fibril formation and promoting the disassembly of pre-formed filaments.

Importantly, baicalein-induced Tau oligomers were reported to be non-toxic in neuronal cell-based models, as demonstrated by cell viability assays performed in Neuro-2a neuroblastoma cells. Moreover, baicalein was shown to mitigate the toxicity associated with preformed Tau aggregates, supporting the relevance of this compound as a potential modulator of pathological protein aggregation in neurodegenerative disorders [133].

Although Aβ42 and α-syn differ in their primary sequences and aggregation kinetics, both are intrinsically disordered proteins that can convert into highly ordered, β-sheet-rich amyloid assemblies. Therefore, these observations are relevant to α-syn, as they indicate that baicalein can destabilize β-sheet amyloid architecture and remodel preformed aggregates. Consistently, Zhu et al. showed that the quinone form of baicalein reacts with lysine residues in α-syn to form a Schiff base, generating soluble oligomers that prevent further β-sheet assembly and therefore α-syn fibrillation [134]. Importantly, baicalein also disaggregates pre-existing fibrils, inducing fragmentation along their length and converting fibrils into monomeric and soluble oligomeric species [134]. Recent computational work showed that baicalein destabilizes wild-type α-syn fibrils by disrupting key stabilizing interactions, including the E46-K80 salt bridge, N- and C-terminal β-sheets, and the inter-protofilament interface. Notably, baicalein also perturbs the structure of α-syn fibrils from familial PD-linked protein mutations, E46K and H50Q, although to different extents and through distinct disruption pathways, suggesting that the disruptive effects of baicalein on α-syn fibrils are polymorphism-dependent [135]. In line with these in vitro observations, baicalein has also been shown to modulate α-syn aggregation in both cellular and in vivo models, including neuronal and non-neuronal cell models. Baicalein markedly reduced the formation of α-syn oligomers and protected SH-SY5Y cells from oligomer-induced toxicity, indicating that its anti-aggregation effects extend to intracellular environments [136]. In rotenone-induced mouse model of PD, baicalein administration prevents the α-syn oligomer accumulation in the midbrain, spinal cord and enteric nervous system, restores neurotransmitter deficits and behavioral impairments. Importantly, these effects occur without changes in α-syn transcription, indicating that baicalein acts primarily by suppressing oligomer formation and promoting the destabilization of existing assemblies [137]. Recent studies have further expanded the therapeutic potential of baicalein by demonstrating its efficacy when administered via advanced nanoformulations. Specifically, brain-targeted nanocarriers generated by combining exosomes from human umbilical cord mesenchymal stem cells with baicalein-loaded nanoliposomes were shown to reduce α-syn fibrillation, interfere with secondary nucleation, and promote the disaggregation of pathogenic assemblies. These hybrids also enhanced cellular uptake and crossed a blood–brain barrier (BBB) model, highlighting their potential as nanotherapeutics for PD [138]. In addition, Zhang et al. demonstrated that baicalein encapsulated in human umbilical cord mesenchymal stem cell-derived exosomes (Exo@Bac) significantly improved cognitive deficits and reduced neuronal damage, Aβ deposition, oxidative stress and neuroinflammation in a rat model of Alzheimer’s disease [139]. These protective effects were associated with enhanced antioxidant defenses, decreased pro-inflammatory cytokine levels, and modulation of AMP-activated protein kinase (AMPK) and nuclear factor-kB (NF-κB) signaling pathways, highlighting the potential of baicalein to protect the nervous system from neurodegeneration driven by amyloid toxicity [139].

#### 3.1.2. Brazilin

Brazilin is a natural homoisoflavonoid extracted from *Caesalpinia sappan* L. with known antioxidant, anti-inflammatory [140] and antibacterial properties [141]. This colorless phenolic compound consists of two aromatic rings linked by a five-membered heterocycle. Upon oxidation of its hydroxyl group to a carbonyl group, it is converted into brazilin, a structurally related chromogenic derivative used as a natural colorant [142]. These structural features, together with the documented bioactivities of brazilin, have generated significant interest in this phytochemical as a modulator of pathological protein aggregation. Experimental evidence has shown that brazilin inhibits Aβ42 fibrillogenesis and remodels mature fibrils, leading to a significant reduction in Aβ42 cytotoxicity [143]. It Inhibits amyloid-β (Aβ) fibrillogenesis through hydrophobic and hydrogen bonding interactions, disrupting the intermolecular interactions required for ordered fibril assembly and promoting the formation of large, unstructured, low-toxicity species.

Brazilin was also able to remodel preformed fibrils, redirecting aggregation into disordered species with a reduced ability to catalyze secondary nucleation, a major source of toxic oligomers. Its ability to cross the BBB supports the therapeutic potential for Alzheimer’s disease. A mechanistic insight into the anti-aggregative activity of brazilin can be gained by examining metal-induced aggregation. Guo et al. demonstrated that brazilin could chelate Zn^2+^ with a physiologically relevant affinity, thereby inhibiting Zn^2+^-mediated Aβ aggregation and reducing cytotoxicity [144]. Notably, brazilin exhibits a greater binding affinity for Aβ than for Zn^2+^, allowing it to compete with the metal ion and prevent Zn^2+^-induced aggregation [144]. Given its ability to remodel pathogenic amyloids, growing attention has also turned to its potential relevance in disorders driven by α-syn. More specifically, in vitro studies have shown that brazilin inhibits α-syn fibrillogenesis by prolonging the nucleation phase of aggregation and reducing the formation of β-sheet-rich fibrils, which are structural transitions required for fibril maturation. In addition to preventing fibrillation, brazilin was found to remodel preformed α-syn fibrils in differentiated PC12 cells by fragmenting mature fibers into smaller, amorphous aggregates with a lower cytotoxicity. Further mechanistic insight from all-atom molecular dynamics simulations revealed that brazilin interacts directly with α-syn oligomeric assemblies. This binding is primarily driven by hydrophobic contacts, with unfavorable polar contributions, and involves three main interaction regions known to influence α-syn aggregation propensity. These interactions destabilize β-sheet structure, increase the solvent exposure of the pentameric assembly, and reduce intermolecular hydrogen bonding, disrupting the fibrillar architecture [145]. A recent study has provided detailed insight into the mechanism by which brazilin modulates α-syn aggregation. Rather than acting on the initial nucleation step, brasilin binds selectively to the compact conformation of monomeric α-syn, thereby stabilizing its unfolded state and reducing the amount of aggregation-competent monomers. In addition, brazilin inactivates pre-existing fibrils by promoting their conversion into large, inert aggregates, reducing the propagation of misfolded α-syn assemblies [146]. The anti-aggregative effects of brazilin extend beyond proteins involved in neurodegenerative diseases. In type II diabetes, brazilin has been shown to inhibit the fibrillogenesis of human islet amyloid polypeptide (hIAPP), a key pathological hallmark of pancreatic β-cell degeneration. Furthermore, brazilin also disassembled pre-existing fibrils into less structured assemblies with low toxicity [147].

Given the multiple anti-aggregative actions of brazilin on α-syn, recent work has focused on the optimization of the pharmacological properties of this phytochemical, reducing its intrinsic limitations. In this context, a novel brazilin derivative, brazilin-7-acetate (B-7-A), has emerged as a promising molecule with improved stability, reduced toxicity and increased efficacy against α-syn aggregation [148].

#### 3.1.3. Curcumin

Curcumin, the main bioactive compound found in the rhizomes of *Curcuma longa* L. [149], is a diarylheptanoid polyphenol recognized for its pharmacological benefits in various pathological contexts, including diabetes, cancer, and neurodegenerative diseases due to its potent anti-inflammatory, antioxidant, anticancerous, immunomodulatory, neuroprotective, and antibacterial activities [150,151,152,153,154]. Curcumin exists in solution as an equilibrium mixture of keto and enol tautomers. Its function is to group curcumin to a wide range of non-covalent interactions with biomolecules. Specifically, the aromatic ring is involved in π–π stacking interactions, whereas the phenolic and keto–enol groups form hydrogen bonds. In addition, due to its structural flexibility, the molecule can adopt a conformation suitable for overall hydrophobic interactions [149]. Through these interactions, curcumin binds various proteins and peptides, thereby modulating their conformation, dynamics and stability, and promoting or inhibiting protein aggregation [155]. Curcumin exerts its inhibitory effects by directly interacting with disordered proteins and modulating the aggregation pathway at multiple levels. Several studies have shown that curcumin can interact with specific structural motifs in Aβ, including the N-terminal region and the central hydrophobic core that drive nucleation and fibril growth, thereby suppressing the fibrillogenesis both in vitro and in vivo [153,156,157]. Curcumin not only interferes with the formation of β-sheet–rich amyloid assemblies but also remodels pre-existing fibrillar structures [158]. In this context, it was demonstrated that curcumin induces marked structural rearrangements in the Asp23–Lys28 salt-bridge region and at the C-terminus of the Aβ fibrils, generating less ordered conformers with reduced stability [159]. Kumaraswamy et al. further revealed that curcumin binds to the core-recognition motif KLVFF of Aβ_1−42_ with micromolar affinity through hydrophobic forces and hydrogen bonding interactions, promoting β-sheet disruption and reducing the cytotoxicity associated with oligomeric and fibrillar Aβ species [160]. Consistently, computational studies showed that curcumin preferentially binds to the 16KLVFFA21 steric-zipper region in an extended conformation, displaying higher binding stability and inducing local β-sheet fluctuations that may redirect Aβ towards less toxic aggregation pathways [161].

Molecular dynamics simulations also revealed that curcumin intercalates among Aβ monomers during primary nucleation and disrupts the formation of an ordered amyloid nucleus by establishing multiple non-covalent interactions, leading to larger, more disordered, and less aggregation-competent early assemblies [162].

Curcumin could prevent the aggregation of α-syn by inhibiting early misfolding events, remodeling oligomeric intermediates, and reducing the stability of preformed fibrillar assemblies, as shown in [112]. Specifically, curcumin interferes with the early stage of α-syn aggregation, breaking down large, insoluble aggregates into smaller, less compact and more soluble species. Singh et al. showed that the phytochemical also modulates the earliest steps of α-syn misfolding by binding selectively to oligomeric and fibrillar species rather than monomers, reducing hydrophobic surface exposure and accelerating their conversion into less toxic fibrillar forms [163]. Mechanistic insights further revealed that curcumin binds strongly to the hydrophobic NAC region of α-syn and completely prevents oligomer and fibril formation by increasing the intramolecular reconfiguration rate and reducing hydrophobic exposure, thereby preventing early association propensity [117]. A molecular dynamics simulation study has also shown that curcumin destabilizes α-syn oligomers by disrupting their β-sheet structure, reducing hydrogen bonds in the main chain and preventing the orderly assembly of NAC structures, thereby inhibiting the progression of oligomers towards fibrillar forms [164].

Several studies suggested that α-syn initiates conformational change and amyloid formation from LLPS, resulting in liquid-like droplets that undergo a liquid-to-solid transition and form an amyloid hydrogel enriched in oligomeric and fibrillar species [165,166,167].

Recent studies collectively demonstrated that curcumin does not alter droplet formation or initial morphology but reduces protein mobility inside the condensates and blocks the liquid-to-solid transition, preventing LLPS-driven amyloid formation [125]. The neuroprotective effects of curcumin are also reflected in its ability to prevent α-syn-mediated neurotoxicity. In SH-SY5Y cells, curcumin has been shown to reduce α-syn-associated toxicity by reducing ROS levels, inhibiting caspase-3 activation, and limiting apoptotic cell death [168]. Using the same in vitro model, Jiang et al. demonstrated that the phytochemical promotes the clearance of pathogenic A53T α-syn, closely associated with hereditary early-onset PD, by restoring macroautophagy through downregulation of the mammalian target of rapamycin (mTOR)/p70S6K pathway, a key negative regulator of autophagy [169]. Curcumin also exerts marked neuroprotective effects in PD models of mitochondrial dysfunction. It was demonstrated that protection against A53T α-syn-induced cell death can be achieved by curcumin through the reduction in intracellular ROS, mitochondrial dysfunction and the activation of apoptosis in a PC12 inducible cell model of PD [170]. In a cellular model of early-onset PD involving knockdown of the PTEN-induced kinase 1 (PINK1) gene, curcumin was found to rescue cell viability, preserve mitochondrial membrane potential and respiratory capacity, and reduce apoptosis, under both basal conditions and in the presence of the toxin paraquat, which is known to induce parkinsonism through mitochondrial dysfunction and oxidative stress induction [171]. Consistently, curcumin counteracts α-syn fibril-induced hexokinase I dissociation, preserving mitochondrial function and limiting ROS generation and oxidative stress [172].

Despite its multi-level actions in limiting α-syn-induced cytotoxicity, curcumin suffers from low permeability, poor bioavailability and instability due to keto-enol tautomerism at physiological pH [173]. To overcome these limitations, curcumin analogues or derivatives and advanced delivery systems have been developed. Notably, 4-arylidene curcumin derivatives have been shown to inhibit α-syn aggregation and promote the disassembly of preformed fibrils [174]. In addition, curcumin glucosides more effectively inhibit α-syn aggregation than native curcumin by promoting a-helical conformations and exhibit improved solubility, bioavailability, and tissue distribution [175]. Considering that nanoparticle delivery systems are widely employed to enhance stability and solubility, curcumin-loaded biomimetic nanomedicines have emerged as a promising strategy to improve biocompatibility, immune evasion, and targeted delivery [176,177,178,179]. In the context of α-syn-driven neurotoxicity, recent evidence indicates that an ROS-responsive nanodrug based on polydopamine-assembled curcumin nanoparticles (PDA-Cur NPs), subsequently functionalized with the brain-targeting RVG29 peptide, enhances brain delivery, scavenges ROS, and prevents α-syn aggregation [180].

#### 3.1.4. Epigallocatechin-3-Gallate

Green tea extract, (-)-epigallocatechin-3-gallate (EGCG), is a well-known antioxidant and anti-inflammatory compound for several diseases [181,182,183,184]. This phytochemical, with hydrophobic (aromatic ring) and hydrophilic (phenolic hydroxyl) moieties, also mediates structural rearrangement of fibrils and exhibits anti-amyloidogenic activity. It interferes with the misfolding and aggregation of disease-linked peptides, like amyloid-beta (Aβ) in Alzheimer’s and α-syn in PD and reduces cellular toxicity [95,185,186,187]. Specifically, EGCG has been shown to reduce α-syn toxicity by redirecting the amyloid fibril aggregation pathway toward non-toxic aggregates. In this context, Ehrnhoefer et al. demonstrated that EGCG interacts with natively unfolded α-syn monomers, preventing the formation of β-sheet–rich, seeding-competent species and inhibiting fibrillogenesis [94]. Instead of allowing amyloid assembly, EGCG redirects α-syn into stable, off-pathway, non-toxic spherical oligomers, acting as a chemical chaperone that modulates the folding and aggregation landscape of the protein, even in the presence of preformed fibrils [94]. It has also been shown that EGCG inhibits α-syn fibrillation in a dose-dependent manner, preventing transition from random coil structure to β-sheet structures by interacting with specific α-syn residues through hydrogen bonding and π–π stacking. This interferes with the formation of the amyloid core and inhibits α-syn-induced cell death [188]. A study investigated the influence of EGCG on α-syn fibril by molecular dynamics simulations. They showed that EGCG disrupts the local β-sheet structure and hydrophobic interactions, stabilizing the inter-protofibril interface and destabilizes the global structure of the α-syn fibrils [189]. In addition, molecular dynamics simulations have revealed that the effects of EGCG on α-syn protofibrils are environment-dependent. Notably, in aqueous solution, EGCG directly destabilizes β-sheet-rich regions of the protofibril, whereas in the presence of lipid membranes, EGCG primarily attenuates protofibril–membrane interactions by binding the C-terminal region of α-syn and forming contacts with membrane headgroups [96]. EGCG remodels mature fibril assemblies into unstructured, non-toxic species. By binding directly to β-sheet-rich α-syn fibrils, EGCG induces conformational remodeling into smaller, amorphous aggregates that are biologically inert. Notably, this process does not involve fibril disassembly into soluble monomers or toxic oligomeric intermediates but instead reflects a reorganization of protein–protein interactions within the fibrillar assembly [95]. In addition, Palhano et al. suggested that the remodeling process involved a mechanism dependent on EGCG auto-oxidation, through interaction with hydrophobic sites within cross-β fibrils as well as covalent interactions (e.g., Schiff base formation) which stabilize remodeled, less toxic aggregates [190]. Interestingly, EGCG has been reported to reduce α-syn toxicity by accelerating fibril formation. In this context, EGCG promotes the conversion of transient, membrane-interactive oligomers into mature amyloid fibrils, thereby depleting the pool of highly toxic species and suppressing membrane disruption [191]. Similarly, Grønnemose et al. demonstrated that α-syn oligomers exist as two interconverting species that differ in the structural organization of their N-terminal region. EGCG modulates this equilibrium through an oxidation-dependent mechanism, making the oligomers less cytotoxic [192].

EGCG has also been reported to reduce the toxicity of α-syn aggregates in vivo. In 1-methyl-4-phenyl-1,2,3,6-tetrahydropyridine(MPTP)-based models of PD, post-treatment with EGCG exerts neuroprotective effects associated with upregulation of the iron-export protein ferroportin in the substantia nigra, leading to reduced nigral iron levels, as well as improved dopaminergic function, motor performance, and decreased oxidative damage [193]. Notably, the neuroprotective properties of EGCG were also demonstrated in the context of the chronic PD progression using a chronic α-syn preformed fibril (PFF)-induced mouse model, in which EGCG was administered prior to disease onset. EGCG pretreatment provided long-term neuroprotection by reducing motor and behavioral deficits, preserving nigrostriatal dopaminergic neurons, decreasing pathological α-syn accumulation, and regulating neuroinflammatory responses [194]. Despite its promising neuroprotective properties, improving the bioavailability of EGCG via nano-formulation strategies may further enhance its therapeutic potential [195,196,197].

In this context, EGCG-functionalized gold nanoparticles have been developed as an effective anti-amyloid strategy, showing strong inhibition of Aβ42 aggregation through direct peptide interactions and significantly attenuating Aβ-induced cytotoxicity [198].

#### 3.1.5. Kaempferol

Kaempferol is a natural flavonol found in many fruits and vegetables, as well as in medicinal plants such as *Ginkgo biloba* [199,200,201]. Growing evidence indicates that kaempferol exerts a series of biological and pharmacological properties, including neuroprotective [202], anti-inflammatory [203], antioxidant [204], antidepressant [205] and antiepileptic [206] effects. Kaempferol has been reported to interfere with the formation of pathogenic protein aggregates [207,208]. Specifically, Kaempferol exerts neuroprotective effects by both activating transcription factor EB (TFEB)-dependent lysosomal biogenesis to promote autophagic clearance of α-syn and directly inhibiting amyloid fibril formation [209]. Moreover, in a transgenic Drosophila model of PD, kaempferol was found to reduce α-syn-induced oxidative stress, apoptosis and dopaminergic neurodegeneration. These protective effects were associated with the enhancement of antioxidant defense systems and the suppression of caspase-dependent apoptosis. Molecular docking further suggested direct interactions between kaempferol and aggregation-prone regions of α-syn, indicating a potential role in inhibiting amyloid formation [210].

#### 3.1.6. Oleuropein

Oleuropein is a phenolic secoiridoid glycoside composed of hydroxytyrosol, elenolic acid, and glucose moiety. It is one of the most abundant bioactive compounds found in the leaves of the olive tree (*Olea europaea* L.). Upon enzymatic hydrolysis, oleuropein loses its glucose residue and is converted into the more lipophilic oleuropein aglycone (OleA), which is considered a major contributor to its biological activity [211]. Oleuropein displays multiple beneficial biological properties, including antioxidant [212], anti-inflammatory [213], anticancer [214], and cardioprotective activities [215]. Oleuropein also exhibits significant neuroprotective effects in several experimental models [216], particularly by interfering with the aggregation of amyloidogenic proteins, including Aβ peptides [217], tau [218] and α-syn [219]. Specifically, OleA exerts strong anti-amyloidogenic activity by engaging regions within the NAC and C-terminal domains of α-syn, reducing hydrophobic interactions that drive amyloid assembly and redirecting aggregation towards stable, non-toxic species. As a result, α-syn assemblies formed in the presence of OleA exhibit attenuated membrane interaction and reduced oxidative stress and cytotoxicity effects. OleA also promotes the remodeling of oligomeric species into inert assemblies [89]. In this context, it was found that hydroxytyrosol strongly inhibits and destabilizes α-syn fibrillation, reducing α-syn–induced cytotoxicity [220]. Molecular dynamics simulations showed that the inhibitory effects of OleA are linked to the reduced hydrophobic interactions between the NAC and C-terminal regions of α-syn, increasing their intramolecular separation and stabilizing the aggregation-incompetent monomeric conformation [221]. Accordingly, a subsequent study identified hydroxytyrosol as a key bioactive metabolite responsible for the anti-amyloidogenic effects of OleA. Mechanistically, hydroxytyrosol interacts with the NAC and C-terminal regions of α-synuclein through both non-covalent hydrophobic interactions and covalent modifications, including methionine oxidation and catechol-mediated adduct formation, altering the conformational landscape of α-synuclein [222]. The neuroprotective potential of olive-derived polyphenols extends beyond hydroxytyrosol to include its metabolites. Notably, the hydroxytyrosol-derived metabolite 4-hydroxy-3-methoxyphenylethanol (MOPET) has been shown to inhibit α-synuclein fibrillization, thereby preventing α-syn-induced neurotoxicity. In addition to its direct anti-aggregative effects, MOPET increases sirtuin 1 (SIRT1) expression under non-aggregating conditions and inhibits SIRT2 expression, a deacetylase known to promote α-syn aggregation and cytotoxicity. Moreover, MOPET significantly upregulated Heat shock protein 70 (Hsp70), a molecular chaperone involved in the refolding and clearance of misfolded α-syn species [223].

In vivo evidence further supports the neuroprotective potential of oleuropein. In rotenone-induced PD mouse models, oleuropein administration significantly improved motor performance and reduced neuronal loss. At the molecular level, oleuropein restored mitochondrial function and calcium homeostasis, two key determinants of α-syn aggregation propensity. Notably, oleuropein rescued BDNF–tropomyosin receptor kinase B (TrkB)–Akt/CREB signaling, which is disrupted by pathological α-syn accumulation and essential for neuronal survival [224].

Effective drug delivery to the brain remains a major challenge in neurodegenerative disorders, with the use of suitable nanocarriers playing a key role in addressing this limitation [225]. Recently, hybrid brain-targeted nanocarriers have been developed by combining exosomes derived from human umbilical cord mesenchymal stem cells with nanoliposomes loaded with oleuropein. This nanosystem has been shown to reduce α-syn fibrillation, interfere with secondary nucleation, and promote the disaggregation of mature fibrils. In addition to its anti-amyloid activity, the hybrid demonstrated strong antioxidant properties attributable to the phenolic compound, effectively scavenging ROS and reducing oxidative stress. Notably, the hybrids exhibited superior cellular uptake and markedly enhanced penetration across an in vitro BBB model compared to nanoliposomes alone, highlighting the therapeutic potential of hybrid nanocarriers in synucleinopathies [226].

#### 3.1.7. Quercetin

Quercetin (3,3′,4′,5,7-pentahydroxyflavone) is a naturally occurring flavonol widely found in many fruits and vegetables. Its structure, characterized by five hydroxyl groups, determines its biological activity and enables the formation of different derivatives [227]. This phytochemical has been shown to exert multiple beneficial biological properties, such as antioxidant [228], anticancer [229], cardioprotective [230], and anti-inflammatory activities [231]. Quercetin also exhibits neuroprotective effects by modulating different signaling pathways. Specifically, it promotes BDNF expression, supporting neuronal survival, synaptic plasticity and cognitive function [232]. In parallel, quercetin promotes mitochondrial biogenesis and maintains the membrane potential by modulating the PI3K/Akt pathway, reducing apoptosis and energy deficits [233]. Additionally, quercetin suppresses neuroinflammation by inhibiting NF-κB signaling and reducing the production of pro-inflammatory cytokines [234]. Finally, it inhibits the amyloid formation of Aβ [235], α-syn [126], and hIAPP proteins [236]. Specifically, quercetin interacts with monomeric α-syn and stabilizes oligomeric species, inhibiting fibril elongation. Moreover, quercetin promotes the disassembly of preformed α-syn fibrils into stable oligomeric species. Oxidized quercetin derivatives further enhance this effect by redirecting the α-syn aggregation pathway [126]. Quercetin also exerts in vivo neuroprotective effects against α-synuclein toxicity through indirect mechanisms. In *C. elegans* models expressing human α-syn, quercetin significantly reduced proteotoxicity and extended lifespan, correlating with a prolonged induction of metallothioneins, key regulators of metal homeostasis and oxidative stress [237]. A recent study using quercetin-loaded nanoemulsions (QNEs) demonstrated improved solubility, stability, and bioavailability of quercetin. More specifically, treatment with QNEs significantly reduced α-syn aggregation in transgenic *C. elegans* expressing human α-synuclein, likely through covalent interactions, consistent with previous reports [126]. Furthermore, QNEs administration was found to improve mitochondrial content, reduce ROS levels and extend the lifespan of α-syn-expressing nematodes. These evidences suggest a multifaceted neuroprotective mechanism involving the attenuation of oxidative stress, preservation of mitochondria and modulation of proteostasis [238].

#### 3.1.8. Resveratrol

Resveratrol (3,4′,5-trihydroxystilbene) is a natural stilbene polyphenol primarily found in grapes and red wine, characterized by two aromatic rings linked by an ethylene bridge and existing as trans- and cis-isomeric forms [239]. Resveratrol exerts multiple biological effects, including antioxidant [240], anti-inflammatory [241], anti-obesity [242], anticancer [243], anti-diabetic [244], cardiovascular [245], and neuroprotective [246] properties.

Initially investigated for its anticancer properties [247], resveratrol has more recently attracted considerable interest in neuroscience due to its robust neuroprotective activity and its ability to activate SIRT1, a NAD^+^-dependent deacetylase [248]. In the context of neuroprotective mechanisms, resveratrol has been shown to reduce toxicity related to α-syn aggregates. Specifically, resveratrol has been shown to attenuate α-syn-induced neurotoxicity through a SIRT1-dependent mechanism. Albani et al. demonstrated that SIRT1 activation by resveratrol reduces oxidative stress and protects neuronal cells from the toxicity of aggregation-prone α-syn species, highlighting a sirtuin-mediated neuroprotective pathway [249]. Notably, SIRT1 activation converges on the AMPK- peroxisome proliferator-activated receptor gamma coactivator 1-alpha (PGC-1α) axis, leading to transcriptional induction of PGC-1α, a master regulator of mitochondrial biogenesis and oxidative metabolism [250]. Given that PGC-1α was deregulated in PD [251], Eschbach et al. proposed a pathogenic link between impaired PGC-1α signaling and α-syn-mediated toxicity [252]. Pharmacological activation of PGC-1α by resveratrol, as well as genetic PGC-1α overexpression, markedly reduced α-syn oligomer formation in a time- and dose-dependent manner, whereas PGC-1α deficiency promoted α-syn oligomerization and neuronal vulnerability. Specifically, restoration of PGC-1α activity attenuated α-syn toxicity by improving mitochondrial function, limiting oxidative stress, and enhancing autophagy-lysosomal clearance [252].

Subsequent studies have supported the direct involvement of resveratrol in the aggregation of α-syn. Specifically, resveratrol has been shown to interfere with the formation of early oligomeric species through transient hydrophobic and π–π interactions, preventing the formation of stable β-sheet-rich fibrils. This interaction redirects the self-assembly of α-syn towards alternative, less toxic pathways, characterized by reduced involvement of the NAC region, increased helical structure and partial destabilization of mature aggregates [253].

Moreover, a recent study has shown that resveratrol modulates α-syn aggregation by inhibiting fibril formation and remodeling mature fibrils into non-toxic assemblies enriched in β-turn conformations, thereby significantly reducing cytotoxicity and ROS production in SH-SY5Y cells. Notably, computational docking analyses suggested that these disruptive effects may involve the ability of resveratrol to bind to specific residues that play a crucial role in maintaining the stability of α-syn fibrils, such as the intra- and inter-peptide E46-K80 and K45/H50-E57 salt bridges at the inter-protofibril interface [254].

Consistent with the anti-amyloid properties reported for resveratrol, its synthetic derivative AM17 was shown to inhibit the aggregation of α-syn monomers and to disassemble n oligomeric and fibrillar species in a copper-independent manner, likely by acting on regions involved in aggregation such as the NAC domain [255].

Accumulating evidence indicates that resveratrol attenuates α-syn pathology through regulation of protein homeostasis and mitochondrial pathways. In MPTP-treated mice, resveratrol activated SIRT1, leading to deacetylation of microtubule-associated protein 1 light chain 3 (LC3), enhanced autophagic flux and promoted clearance of cytosolic α-syn [100]. In parallel, resveratrol limits mitochondrial α-syn accumulation by downregulating the voltage-dependent anion channel 1 (VDAC1) and preventing opening of the mitochondrial permeability transition pore, thereby preserving mitochondrial function and reducing pro-apoptotic signaling. Consistent with these mechanisms, dietary resveratrol mitigated α-syn-associated toxicity and improved neuronal function in a transgenic Drosophila melanogaster model expressing human α-syn, in association with reduced oxidative stress and enhanced antioxidant defenses. Furthermore, molecular docking analyses revealed that resveratrol directly interacts with α-syn through hydrogen bonding at residues involved in protein–protein interactions and aggregation [116].

### 3.2. Alkaloids

Alkaloids are a class of nitrogen-containing molecules produced from amino acids that are primarily found in plants [256], that exhibit a wide range of biological activities, including antioxidant [257], anti-inflammatory [258] and antidepressant [259] effects.

Alkaloids are structurally classified as heterocyclic or non-heterocyclic compounds based on the position of the nitrogen atom. Heterocyclic alkaloids incorporate nitrogen within the core ring system and typically derive from the decarboxylation and modification of amino acid precursors, such as L-tyrosine, L-phenylalanine, L-ornithine, L-tryptophan, L-lysine and L-histidine. In contrast, non-heterocyclic alkaloids contain nitrogen in an aliphatic side chain [260,261]. In the context of neurodegenerative diseases, alkaloids exert neuroprotective effects through different mechanisms, including the modulation of oxidative stress [262], neuroinflammation, mitochondrial homeostasis [263], and proteostasis [264]. In the context of alkaloid-mediated proteostatic regulation, caffeine and nicotine represent two of the most well-characterized compounds for their ability to modulate α-syn-associated toxicity [265,266]. The effects of selected alkaloids and the experimental methods used to evaluate their impact on α-syn conformational states are summarized in Table 1, while experimental models and concentration ranges are detailed in Supplementary Table S1.

Despite sharing neuroprotective properties, caffeine and nicotine differ markedly in their chemical structures, a feature that likely underlies their distinct mode of interaction with α-syn. Caffeine is characterized by a rigid, planar purine scaffold with limited protonation at physiological pH, favoring transient and low-affinity contacts with the protein. In contrast, nicotine possesses a more flexible bicyclic structure with a protonatable nitrogen [267], enabling stronger interactions with aggregation-prone α-syn species. Consistent with these structural differences, caffeine predominantly mitigates α-syn-related toxicity through indirect mechanisms, by modulating synaptic activity, neuroinflammatory response and protein homeostasis, largely via antagonism of adenosine A_2_A receptors [268]. In vitro and in a yeast PD model, it was shown that caffeine accelerates α-syn aggregation kinetics while redirecting self-assembly towards amorphous or structurally altered aggregate species with reduced cytotoxicity. Biophysical analyses indicated that caffeine interacts with the protein through transient and low-affinity interactions, inducing conformational rearrangements that qualitatively alter the aggregate properties of α-syn [269].

In vivo, chronic caffeine administration confers neuroprotection in α-syn-based PD model by reducing phosphorylated Ser129-positive inclusions, neuroinflammation and neuronal apoptosis. Notably, these effects are linked to restoration of proteostasis via activation of macroautophagy and chaperone-mediated autophagy, rather than direct inhibition of fibril formation [265]. Consistently, single-molecule nanopore analysis further supports this evidence, revealing a transient interaction between caffeine and monomeric α-syn, supporting the notion that its neuroprotective effects arise primarily from modulation of aggregate quality [270].

In contrast to caffeine, nicotine has been shown to interact more directly with α-syn and to modulate its aggregation at multiple levels. At the protein level, nicotine binds monomeric α-syn and induces conformational rearrangements that delay nucleation, thereby prolonging the lag phase and slowing the formation of soluble oligomeric species. This early interference with aggregation distinguishes nicotine from caffeine, which primarily affects late-stage aggregate properties [271]. Consistently, Ono et al. demonstrated that nicotine inhibits the formation of α-syn fibrils from monomers and destabilizes preformed fibrils, through interactions mediated by its pyrrolidine moiety [272]. However, these effects occur at concentrations significantly higher than those safely achieved in vivo, suggesting that the predominant mechanism underlying the neuroprotective action of nicotine is not direct inhibition of α-syn assembly [272]. Instead, converging evidence supports a model in which nicotine primarily exerts neuroprotection through receptor-mediated pathways. Specifically, nicotine primarily targets neuronal nAChRs, with high affinity for the α4β2 and α7 subtypes [273,274], whose modulate calcium-dependent signaling cascades that regulate neuronal survival, synaptic activity, mitochondrial function, and neuroinflammatory responses [275,276,277,278]. Activation of β2-containing nAChRs within dopamine D3 receptor-nAChR heteromeric complexes promotes PI3K-dependent pro-survival signaling and restores ubiquitin–proteasome system activity, facilitating α-syn clearance and reducing aggregation-associated toxicity [266]. Consistently, in α-syn preformed fibril-based models, nicotine activated α7 nicotinic acetylcholine receptors to inhibit pathological α-syn accumulation and Ser129 phosphorylation. This effect is associated with reduced hippocampal neuronal apoptosis, preservation of neurogenesis and attenuation of aggregation-associated toxicity, likely mediated through suppression of glial activation and engagement of PI3K-AKT signaling [279]. Another recent study reinforced this evidence by showing that neuroprotection mediated by nicotine in synucleinopathy models depends critically on α4β2 nAChR signaling. Notably, the activation of α4β2 nAChRs reduces α-syn aggregation, phosphorylation and neurotoxicity in both neuronal cultures and mouse models, identifying this receptor subtype as a central effector of nicotine-induced protection against α-syn-driven pathology [280].

### 3.3. Ginsenosides

Ginsenosides are steroid-like compounds found in *Panax* plants [281]. *Panax ginseng* is a well-known medicinal plant that has been used in East Asia for over two thousand years to treat various conditions [282]. They exert a variety of neuroprotective effects by acting on oxidative, inflammatory, apoptotic, mitochondrial and proteostatic pathways [283,284,285]. Unlike polyphenols, which often act directly on misfolded protein assemblies, ginsenosides have been shown to modulate α-syn pathology through indirect pathway-mediated mechanisms, primarily by remodeling the cellular environment that governs α-syn homeostasis. In this context, ginsenoside Rg1 has been shown to enhance motor function and protect nigrostriatal dopaminergic neurons in a chronic MPTP/probenecid mouse model [286]. Several mechanisms have been hypothesized to explain the decreased accumulation of oligomeric and Ser129-phosphorylated α-syn species as well as Lewy body-like inclusions in the substantia nigra in this mouse model. These beneficial effects of ginsenoside Rb1 is most likely mediated by the suppression of neuroinflammatory signaling and the restoration of cellular homeostasis rather than from the direct inhibition of α-syn aggregation. Ginsenoside Rg1 has been shown to exert robust cytoprotective actions in PC12 cells undergoing apoptosis induced by dopamine. This is achieved by attenuating oxidative stress, preserving mitochondrial integrity, and inhibiting apoptotic signaling cascades [287]. Furthermore, RG1 pre-treatment showed protection against H_2_O_2_-induced neuronal apoptosis through myosin IIA-actin-related cytoskeletal reorganization in PC12 cells and primary neurons [288]. In an in vivo animal model of PD, Rg1 treatment significantly alleviated both motor and non-motor PD symptoms, attenuated the degeneration of dopaminergic neurons and reduced pathological αα aggregation in the striatum and substantia nigra pars compacta (SNpc) enhancing protein autophagy [289]. In addition to their antioxidant and cytoprotective properties, ginsenosides exhibit powerful anti-inflammatory activity highly relevant to α-syn-related neurodegeneration.

The neuroprotective and anti-inflammatory properties of ginsenoside Rb1 were investigated in a lipopolysaccharide-induced rat PD model. The results showed that Rb1 attenuates dopaminergic neurodegeneration by suppressing the activation of microglia and the resulting neuroinflammation mediated by NF-κB, as well as reducing the pathological accumulation of α-syn in the nigrostriatal system [290].

While most ginsenosides primarily modulate the cellular determinants of α-syn toxicity, converging evidence indicates that ginsenoside Rb1, a principal compound in ginseng, directly engages α-syn conformational intermediates. Biophysical and cellular studies demonstrate that ginsenoside Rb1 can inhibit α-syn fibrillation and toxicity in vitro, as well as disaggregate preformed fibrils and block α-syn seeded polymerization. This may be achieved by binding to soluble non-toxic oligomers with no β-sheet content, making it susceptible to proteinase K digestion [291]. This selective interaction stabilizes protease-sensitive, non-pathogenic α-syn species and prevents their conversion into fibrillar assemblies associated with cytotoxicity.

Notably, Rb1 treatment has also been shown to restore MPTP-reduced α-syn expression to normal levels in the CA3 region of the hippocampus, increasing monomeric rather than oligomeric α-syn expression through the regulation of the trans-synaptic α-synuclein/PSD-95 signaling axis [292].

Taken together, these findings suggest that ginsenosides primarily influence cellular pathways that subsequently affect α-syn aggregation and toxicity indicating a multi-target, systems-level mode of action. The clinical investigation of ginsenosides for the treatment of neurodegenerative diseases is limited, primarily due to their bioavailability. The developments recent advances in drug delivery strategies provide robust scientific and technological foundations for the potential clinical application of ginsenosides in the treatment of neurodegenerative diseases [293,294,295].

The effects of ginsenoside Rb1 and the experimental methods used to evaluate their impact on α-syn conformational states are summarized in Table 1, while experimental models and concentration ranges are detailed in Supplementary Table S1.

**Table 1:** Natural compounds targeting distinct α-syn conformational states in PD.

Nutraceuticals	Effects	α-Syn Stage Targeted	Experimental Methods	References
**BAICALEIN** 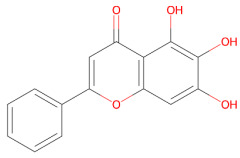	Forms covalent Schiff-base adducts with Lys residues (quinone form)	Primary nucleation	Mass spectrometry analysis	[134]
	Stabilizes soluble, non-toxic oligomers	Oligomeric stage	ThT fluorescence assay; Atomic Force Microscopy (AFM); measurements; Size Exclusion (SEC) HPLC measurements; Circular Dichroism (CD) spectroscopy	[134]
			Transmission Electron Microscopy (TEM)	[134,137]
			Immunofluorescence (IF); Western Blot analysis	[137]
	Disrupts β-sheet assembly	Primary nucleation; oligomeric stage	Molecular dynamics simulations	[135]
	Disaggregates mature fibrils	Fibrillar stage	ThT fluorescence assay; AFM measurements	[134]
			TEM	[134,137]
	Destabilizes fibril architecture by disrupting E46-K80 salt bridge and the protofilament interface	Secondary nucleation	Molecular dynamics simulations	[135]
	Polymorphism-dependent fibril remodeling	Fibril maturation stage	Molecular dynamics simulations	[135]
**BRAZILIN** 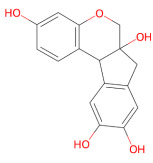	Inhibits α-Syn fibrillogenesis by binding aggregation-competent monomers and oligomers	Primary nucleation; oligomer stage	Molecular dynamics simulations; ThT fluorescence assay	[145]
			Native electrospray ionization-ion mobility mass spectrometry (native ESI–IM–MS)	[145,146]
			CD spectroscopy	[146]
	Disrupts β-sheet-rich fibrils and converts them into large, inert aggregates	Fibrillar stages	ThT fluorescence assay	[145,146]
			Molecular dynamics simulations	[145]
			AFM measurements	[145,146]
			Filter Retardation Assay (FRA); TEM; SDS-PAGE	[146]
	Reduces seeding competence	Secondary nucleation	TEM; ThT fluorescence assay; Real-Time Quaking-Induced Conversion (RT-QuIC)	[146]
**CURCUMIN** 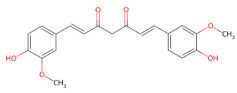	Binds the NAC hydrophobic region via non-covalent interactions	Primary nucleation	NMR spectroscopy; Size-exclusion chromatography (SEC)	[163]
			Molecular dynamics simulations	[88]
	Inhibits β-sheet formation	Primary nucleation; oligomeric stage	CD spectroscopy	[117,163]
			ThT fluorescence assay	[88,117,125,163]
			TEM	[88,125]
	Remodels oligomers into less toxic species	Oligomeric stage	Western blot analysis	[112]
			Native PAGE	[88]
	Destabilizes preformed fibrils	Fibrillar stages	Congo Red binding assay; AFM measurements; SDS-PAGE	[163]
			ThT fluorescence assays	[88,125]
			TEM	[88,125]
	Blocks LLPS-driven liquid-to-solid transition	LLPS-mediated aggregation	Fluorescence Recovery After Photobleaching (FRAP)	[125]
			Confocal microscopy; Differential Interference Contrast (DIC) microscopy	[88]
**EPIGALLOCATECHIN-3-GALLATE (EGCG)** 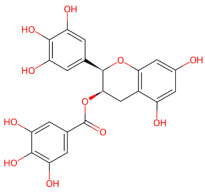	Binds unfolded α-syn	Primary nucleation	NMR spectroscopy	[94,188]
			ThT fluorescence assay	[95]
	Modulates β-sheet structure	Primary nucleation; oligomeric stage	ThT fluorescence assay	[94,95,188]
			CD spectroscopy	[94,95,188,191]
			Fourier Transform Infrared (FTIR) spectroscopy	[191]
	Redirects aggregation toward less toxic oligomers	Oligomeric stage	ThT fluorescence assay	[94,188]
			AFM measurements	[95,188,191]
			TEM	[95,188,191]
			Filter Retention Assay	[95]
	Remodels mature fibrils into amorphous, non-toxic aggregates	Fibril maturation stage	TEM	[94,95,188]
			AFM measurements	[95,188]
			CD spectroscopy; ThT fluorescence assay	[95]
**KAEMPFEROL** 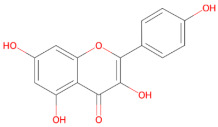	Inhibits α-syn fibril formation	Fibril formation stage	ThT fluorescence assay; TEM	[209]
			Molecular docking analysis	[210]
	Promotes autophagic clearance of α-syn	Proteostasis	Western blot analysis; DAPRed staining qRT-PCR	[209]
**OLEUROPEIN AGLYCONE** 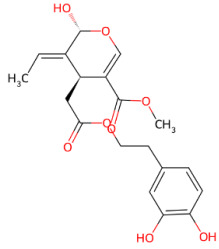	Binds directly to monomeric α-synuclein at the N-terminal region	Primary nucleation	Molecular docking; Molecular dynamics simulations	[221]
	Redirects aggregation toward non-toxic, off-pathway oligomeric species	Oligomeric stage	ThT fluorescence assay	[89,219,222]
			Dynamic Light Scattering (DLS)	[89]
			TEM	[89,219,222]
			Size Exclusion Chromatography-Multi-Angle Light Scattering; CD spectroscopy; SDS-PAGE	[219]
	Inhibits the interaction of α-synuclein aggregates with the cell membranes	α-syn aggregate-membrane interaction	Confocal microscopy	[89,222]
			Förster Resonance Energy Transfer (FRET) analysis; Calcein release assays	[219]
**QUERCETIN** 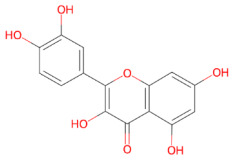	Direct interactions with α-syn	Primary nucleation	UV–Vis spectral titration; ThT fluorescence assays; Light scattering measurements	[126]
	Inhibits fibril elongation	Fibril maturation stage	ThT fluorescence assays; Light scattering measurements	[126]
	Disassembles preformed fibrils	Fibril maturation stages	ThT fluorescence assays; Light scattering measurements; AFM measurements; SEC-HPLC	[126]
	Reduces α-syn toxicity	α-syn-associated toxicity	GFP fluorescence assays	[237]
**RESVERATROL** 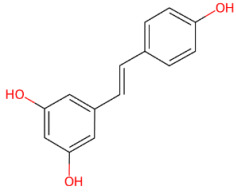	Interacts with aggregation-prone regions	Primary nucleation	Hydrogen-deuterium exchange mass spectrometry (HDX-MS)	[253]
	Reduces the involvement of the NAC region in aggregation	Oligomeric stage	HDX-MS	[253,254]
			Kinetic modeling	[254]
	Redirects aggregation toward off-pathway, non-β-sheet oligomeric species.	Oligomeric stage	CD spectroscopy	[253,254]
			TEM	[254]
	Remodels mature fibrils	Fibrillar stage	TEM	[253]
			ThT fluorescence assays; AFM measurements; CD spectroscopy; SDS-PAGE	[254]
**CAFFEINE** 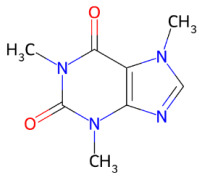	Interacts transiently with monomeric α-syn outside the NAC region	Primary nucleation	ThT fluorescence assay; RP-HPLC analysis	[269]
			CD spectroscopy	[269,270]
			Isothermal titration calorimetry (ITC); NMR spectroscopy	[270]
	Redirecting self-assembly toward low-toxicity aggregates	Oligomeric stage	ThT fluorescence assay; Anilinonaphthalene-8-sulphonic acid (ANS) assay; Dynamic light scattering (DLS) analysis; TEM; Filter retardation assay	[269]
**NICOTINE** 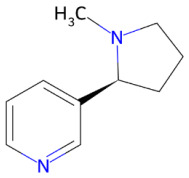	Binds monomeric α-syn	Primary nucleation	Fluorescence quenching assay; CD spectroscopy; RP-HPLC analysis	[271]
	Delays nucleation and slows the formation of soluble oligomers	Oligomeric stage	ThT fluorescence assay; ANS fluorescence assay; DLS analysis; TEM	[271]
	Inhibits fibril formation	Fibrillar stage	ThT fluorescence assay	[271,272]
	Destabilizes preformed fibrils	Fibrillar stage	ThT fluorescence assay; AFM measurements	[272]
**GINSENOSIDE Rb1** 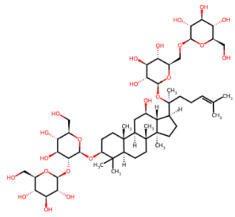	Directly binds soluble non-β-sheet α-syn oligomers	Oligomeric stage	SEC	[291]
	Inhibits fibrillation	Fibril formation stage	ThT fluorescence assay; TEM; Congo Red binding assay; Proteinase K digestion assay	[291]
	Disaggregates preformed fibrils	Fibrillar stage	ThT fluorescence assay; TEM	[291]
	Blocks seeded polymerization	Secondary nucleation	TEM; Seeding polymerization assay	[291]

## 4. Clinical and Translational Perspectives: Current Limitations and Future Directions

### 4.1. Clinical Evidence Supporting Nutraceutical Relevance in Modern and Traditional Alternative Medicine

Traditional systems of medicine, including Ayurveda and Traditional Chinese Medicine (TCM), have long recommended consuming specific foods and botanicals for their potential clinical use in promoting health [296]. Robust cumulative clinical evidence from modern epidemiology, dietary patterns, and traditional medical practices supports the clinical relevance of several nutraceuticals discussed in this review. although they remain largely observational and non-disease-modifying in nature. Large prospective cohort studies provide compelling epidemiological evidence that caffeine, a purine alkaloid, extensively discussed in this review, is linked to a reduced risk of PD. In population-based studies, caffeine exposure is primarily assessed through coffee consumption, as coffee is the main dietary source of this compound. Consistent evidence suggests that coffee intake is inversely related to PD incidence in a dose-dependent manner [297] and appears to delay the age-at-onset of PD rather than reducing overall disease risk [298]. A prospective cohort study validated the protective effect of caffeine on PD risk and further confirmed the causal role of caffeine by analyzing biosamples before PD diagnosis [299]. In a Finnish prospective cohort followed for over 22 years, higher coffee consumption was associated with a markedly reduced PD risk, with the strongest effect observed in individuals consuming ≥10 cups per day [300]. Similar findings were consistently reported in large prospective analyses by Ascherio et al., who combined data from the Health Professionals Follow-up Study and the Nurses’ Health Study [301]. This analysis demonstrated that caffeine intake, rather than other coffee constituents, was inversely associated with PD risk and no protective effect was observed for decaffeinated coffee [301]. Taken together, these studies establish the strong epidemiological relevance of exposure to caffeine derived from coffee intake in relation to PD. At the same time, however, they highlight the gap between population-level associations and molecular target validation.

In addition to caffeine, there is also a growing body of clinical evidence supporting the importance of tea-derived polyphenols, particularly catechins such as EGCG, in delaying the onset of PD or preventing its progression. In epidemiological studies, chronic exposure to these polyphenols is commonly acquired through tea consumption, which is a widespread dietary habit and a long-standing traditional practice, especially in East Asian cultures. A study aimed at examining the relationship between tea consumption and PD risk in the Chinese population showed a dose-dependent protective effect [302]. A meta-analysis of observational studies involving over 1400 PD cases revealed that tea consumption is associated with a reduced risk of PD, with no apparent dose-response relationship [303]. Similarly, a population-based case-control study of people in the United States reported a significantly lower PD risk among individuals consuming two or more cups of tea per day, an effect that remained significant after adjustment for smoking and coffee intake and was not solely attributable to total caffeine consumption [304]. These observations therefore suggest that bioactive compounds in tea beyond caffeine may contribute to the epidemiological association. This interpretation is supported by reviews on tea polyphenols which identify catechins, particularly EGCG, as the major bioactive constituents of green tea, able to exert antioxidant, iron-chelating and neuroprotective activities that are relevant to neurodegenerative disorders [305]. Furthermore, a study examined the association between tea consumption and the risk of incident PD among nearly 30,000 Finnish adults with no history of PD, suggesting that three or more cups of tea intake per day is associated with a lower risk of PD [306]. Although causal inferences cannot be drawn, the clinical plausibility of tea-derived nutraceuticals is supported by the convergence of epidemiological data and traditional use.

TCM applied complex herbal formulations that often combine several herbs, providing a broader framework from which the multiple nutraceuticals discussed in this review originate. These long-standing medicinal practices continue to be widely used in modern clinical settings. Although mechanistic reviews emphasize their proposed multi-target actions on prevention and treatment of PD, direct disease-modifying effects in humans have not yet been conclusively demonstrated [307,308]. Nevertheless, clinical relevance is supported by randomized, placebo-controlled studies performed in a blind manner for 13 weeks, demonstrating that multi-herbal TCM formulations can improve activities of daily living and non-motor symptoms in PD patients, with good tolerability and without modification of standard dopaminergic therapy [309]. In a recent multicenter, randomized, double-blind, placebo-controlled trial, the long-lasting efficacy of Pingchan granules (PCG), which are composed of nine herbs, was demonstrated in reducing motor symptoms and enhancing mobility functions [310]. Furthermore, Hu et al. used a modified Pingchan formula to evaluate the therapeutic effect at different stages of PD [311]. In this multicenter, randomized, double-blind, placebo-controlled clinical trial, they demonstrated a delay in disease progression and an improvement in the complications caused by Western medicine as well as an improvement in patients’ quality of life [311].

These findings demonstrate that there is a body of clinical evidence from traditional medical systems supporting the clinical plausibility of nutraceuticals and nutraceutical-rich interventions in PD. However, they also emphasize the requirement for pharmacokinetically optimized, biomarker-driven clinical studies to bridge the gap between population-level associations and molecular mechanisms.

### 4.2. Clinical and Translational Evidence Across Protein Misfolding Disorders

**Figure 3:**
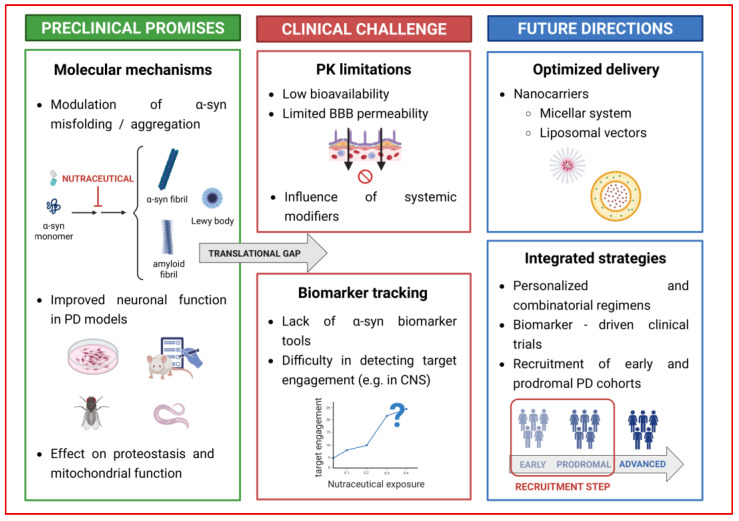
Preclinical promises clinical challenges and future directions for nutraceutical modulation of α-syn pathology. Preclinical studies report multiple effects of nutraceuticals in experimental systems, including direct modulation of α-syn misfolding and aggregation, evidence of neuroprotective effects in cellular and animal PD models, and changes in proteostasis and mitochondrial function (**left** panel). Clinical translation is limited by pharmacokinetics (PK) and systemic factors, including low bioavailability, limited blood–brain barrier (BBB) penetration, systemic modulation of exposure and response, and limited assessment of target engagement in the central nervous system (CNS) (**central** panel). Future directions highlight optimized delivery strategies, together with biomarker-guided clinical trials, combination of interventions, and studies in early and prodromal PD cohorts (**right** panel).

Although clinical trials directly targeting α-syn pathology in PD remain limited, important insights can be drawn from studies of related protein misfolding disorders. These conditions share convergent pathogenic mechanisms, including aberrant protein conformation, aggregation, and clearance impairment. Such cross-disease observations help to clarify the major challenges in translating nutraceutical α-syn modulators into meaningful clinical benefits for PD. These challenges include poor pharmacokinetics, insufficient central nervous system (CNS) penetration and the lack of sensitive biomarkers capable of detecting target engagement in vivo (Figure 3). Preclinical evidence clearly shows that several nutraceuticals can modulate α-syn conformational dynamics, inhibit oligomer formation and stabilize non-toxic assemblies. However, despite these mechanistic advances, demonstrating the translation of such molecular effects into clinical benefit remains difficult to demonstrate, as illustrated by the available human studies. In this context, curcumin is a prime example of the translational gap. In idiopathic PD, curcumin supplementation has been shown to reduce levels of phosphorylated α-syn in skin nerve fibers, compared to untreated patients who showed increased skin aggregates. This rare in vivo evidence of direct α-syn modulation in humans and reinforces the mechanistic plausibility established in preclinical models [312]. Further evidence of curcumin’s biological activity comes from clinical studies of other proteinopathies, where the use of advanced biomarker approaches allows for a more accurate assessment of its effects in vivo. Small et al. conducted a 2-(1-{6-[(2-[F-18]fluoroethyl)(methyl)amino]-2-naphthyl}ethylidene)malononitrile positron emission tomography (FDDNP-PET) scan analysis in an 18-month, double-blind, placebo-controlled trial involving middle-aged and older adults who were not experiencing symptoms of dementia [313]. The results showed that a bioavailable curcumin formulation produced measurable reductions in the binding of amyloid and tau in the amygdala and hypothalamus, demonstrating how molecular imaging can reveal target engagement even when there is no clear clinical improvement [313]. These findings highlight the importance of sensitive biomarkers when evaluating the therapeutic potential of nutraceuticals and imply that similar tools are needed to meaningfully assess α-syn-directed interventions (Figure 3). Despite evidence of biological activity, robust clinical efficacy has not been consistently observed. Ghodsi et al., demonstrated that no significant improvement in motor symptoms was observed in a randomized, triple-blind, placebo-controlled trial evaluating curcumin as adjunct therapy in patients with PD, highlighting the persistent discrepancy between in vitro studies and functional outcomes [314]. This discrepancy is commonly attributed to the limited bioavailability of curcumin and its poor penetration of the CNS, which likely prevents the compound from achieving the concentrations required to translate its mechanistic effects into measurable clinical benefit. A similar pattern emerges with EGCG, which has also failed to demonstrate a therapeutic effect in patients affected by an α-synucleinopathy, such as MSA [315]. These negative outcomes likely reflect its limited BBB penetration rather than a lack of intrinsic anti-aggregation activity [316].

### 4.3. Barriers to Clinical Translation and Future Perspectives

Collectively, these clinical trajectories reveal a consistent translational landscape in which nutraceuticals often exhibit biochemical activity or biomarker evidence but fail to translate these effects into significant clinical benefits. Pharmacokinetic constraints represent a unifying barrier. For example, polyphenols such as curcumin and EGCG are characterized by poor aqueous solubility, rapid metabolic clearance, and limited permeability across the BBB [175]. Accordingly, advanced delivery strategies, including nanocarriers, micellar systems, and liposomal vectors, have been developed to enhance CNS exposure and have demonstrated improved efficacy in preclinical models [158,176,179,196,197]. In line with this approach, curcumin nanoemulsions have been shown to have significantly greater neuroprotective effects than free curcumin in experimental models of PD [317]. Limitations in the design of clinical trials, beyond the specific pharmacology of the compound, further hinder translation. The incorporation of molecular biomarkers, such as CSF α-syn species [318,319], is essential to confirm target engagement and to distinguish true biological activity from pharmacokinetic failure. Moreover, intervention at the early or prodromal stages of PD is necessary to observe disease-modifying effects, given that later stages are characterized by irreversible neurodegenerative processes [320]. Several nutraceuticals discussed in this review, including quercetin, oleuropein, baicalein, brazilin, kaempferol, and ginsenoside Rb1, exhibit compelling preclinical activity against α-syn aggregation, mitochondrial dysfunction, oxidative stress, and neuroinflammation. However, to our knowledge, none of these compounds have been evaluated in clinical trials for PD or other synucleinopathies, nor have any human studies directly assessed their effects on α-syn species in CSF, blood, or peripheral tissues.

Taken together, these findings suggest that pharmacokinetic limitations, inadequate CNS delivery, suboptimal trial design, and systemic modifiers collectively restrict the clinical translation of nutraceutical α-syn modulators. Therefore, future research should prioritize optimized delivery platforms, stage-appropriate and biomarker-driven clinical trials, and rational combinatorial strategies to fully evaluate the neuroprotective potential of nutraceuticals in PD and other synucleinopathies (Figure 3).

## 5. Clinical Trials and Development of Nutraceuticals in PD and CNS Disorders: Implications for α-Syn Research

Building on the translation restrictions described in Section 4, we provide a summary of key clinical trials and clinical development efforts involving nutraceuticals in the treatment of PD and other CNS disorders. The aim is to highlight recurrent translational challenges and inform future drug discovery strategies focused on α-syn.

Firstly, large-scale clinical trials conducted directly in PD have repeatedly revealed a significant discrepancy between the strong preclinical rationale and the demonstrated clinical efficacy. Evidence derived from other α-synucleinopathies consistently supports these conclusions.

A prospective, non-randomized study, reveals that curcumin, a natural polyphenolic compound widely used in traditional medicine, may offer modest benefits as an adjunct therapy in PD when administered in formulations designed to enhance its bioavailability [312]. In this clinical study, long-term curcumin supplementation, using a bioavailable phytosomal formulation, was associated with a slower deterioration of motor function and improvement of specific non-motor symptoms in patients with idiopathic PD, whereas, on the contrary, untreated patients showed progressive clinical worsening. Notably, this study provided rare in vivo biomarker evidence of α-syn modulation, as curcumin-treated patients exhibited reduced phosphorylated α-syn aggregates in skin nerve fibers, particularly in early-stage disease. Although limited by sample size and non-randomized design, this study represents the most direct clinical evidence to date of a link between a naturally derived nutraceutical and α-syn-related biomarker changes in humans. In this context, Ghodsi et al. conducted a pilot randomized, triple-blind trial focusing primarily on motor outcomes and quality of life over nine months, using curcumin formulated in the form of a nanomicelle [314]. Despite preclinical evidence suggesting that curcumin might be a potential neuroprotective agent, the supplementation did not produce significant improvements in overall motor function or quality of life [314]. Similarly, a randomized, double-blind, placebo-controlled trial focusing on non-motor outcomes demonstrated that the nanomicelle curcumin supplement had no significant effect on the fatigue severity of PD patients [321].

Epigallocatechin gallate (EGCG) is one of the most promising nutraceuticals with direct anti-α-syn aggregation activity to be clinically investigated. In the randomized, double-blind, placebo-controlled PROMESA trial conducted in patients with multiple system atrophy, EGCG failed to slow disease progression despite strong preclinical anti-aggregation rationale. Moreover, although exploratory Magnetic Resonance Imaging (MRI) analyses indicated reduced regional brain atrophy in EGCG-treated patients, these structural changes did not result in any measurable clinical benefits. Crucially, dose-limiting hepatotoxicity constrained maximal systemic exposure, likely preventing CNS target engagement, while the absence of direct molecular biomarkers of α-syn further limited interpretation of the negative clinical outcome [322]. Notably, subsequent analyses following the PROMESA trial clarified that negative outcomes do not necessarily indicate an absence of intrinsic anti-aggregation activity in polyphenols. Instead, they emphasize translational limitations, such as the poor absorption of the dietary polyphenols, their rapid metabolic clearance and limited blood–brain barrier penetration, which frequently results in brain concentrations below therapeutic thresholds, and highlight the absence of sensitive biomarkers of target engagement [323]. Taking together, these observations draw attention to pharmacokinetic and delivery constraints as the main obstacles to the clinical effectiveness of nutraceuticals in CNS disorders and argue for nutraceuticals to be repositioned as molecular scaffolds for drug discovery, rather than as ready-to-use therapeutics [316]. In addition, evidence from clinical trials conducted in Alzheimer’s disease provides an instructive comparison, illustrating how pharmacokinetic optimization and biomarker integration can overcome these limitations to same extent. In a long-term randomized, double-blind, placebo-controlled trial involving non-demented middle-aged and older adults, administration of a highly bioavailable curcumin formulation was associated with improvements in memory and attention as well as reduced brain amyloid and tau accumulation, as assessed by FDDNP-PET molecular imaging. Notably, these findings provide direct in vivo evidence of CNS target engagement when bioavailability and delivery are optimized [313]. By contrast, a large phase II randomized trial of high-dose resveratrol in patients with Alzheimer’s disease demonstrated the presence of the compound and its metabolite in the cerebrospinal fluid, confirming blood-brain barrier penetration, however it failed to produce meaningful clinical benefit despite modulation of selected CSF biomarkers [324]. Finally, these convergent clinical experiences across PD, α-synucleinopathies, and other CNS disorders demonstrate that CNS penetration alone is insufficient to ensure therapeutic efficacy. Therefore, the future clinical development of nutraceuticals targeting α-syn should prioritize optimized exposure, careful selection of disease stage, and the incorporation of sensitive biomarkers of target engagement. Taken together, these considerations highlight the importance of formulation-driven strategies and biomarker-guided clinical trial designs as essential prerequisites for the successful translation of nutraceutical-derived compounds into meaningful drug discovery efforts focused on α-synuclein.

## 6. Conclusions

Nutraceuticals have emerged as promising support to conventional therapies, with the potential to reduce treatment-related toxicity by utilizing the biological activity of naturally derived compounds. Accordingly, growing scientific interest has been directed toward elucidating their potential as stand-alone or supportive interventions that modulate key disease-related pathways. This review highlights current evidence on nutraceutical strategies aimed at modulating the conformational landscape of α-syn and related pathogenic processes. A substantial body of preclinical data from both in vitro and in vivo studies demonstrates that a range of natural compounds, including polyphenols, alkaloids, and ginsenosides, can interfere with α-syn misfolding and aggregation, stabilize non-toxic assemblies, preserve mitochondrial function, and mitigate oxidative stress and neuroinflammation, all of which are key processes in synucleinopathies and neurodegenerative disease. Overall, these findings support a biologically plausible rationale for targeting α-syn-driven pathology using nutraceuticals. Despite this strong mechanistic basis, clinical translation remains markedly limited. Currently, only a small subset of compounds, namely curcumin, EGCG, resveratrol, caffeine, and nicotine, have been evaluated in human studies, and none have demonstrated consistent disease-modifying effects or robust modulation of α-syn biomarkers in vivo. In contrast, several nutraceuticals with compelling preclinical activity, including oleuropein, baicalein, brazilin, kaempferol, and ginsenoside Rb1, remain untested in clinical settings, underscoring a substantial gap between experimental promise and clinical validation. Progress in this field will require a shift towards translationally informed study designs, with particular focus on optimized formulations to enhance brain bioavailability, the incorporation of sensitive α-syn biomarkers to confirm target engagement, and the recruitment of early or prodromal PD populations. In conclusion, although nutraceuticals represent a biologically plausible class of α-syn modulators, their clinical potential remains largely unrealized. Overcoming pharmacokinetic limitations, integrating molecular biomarkers, and adopting personalized and combinatorial strategies will be essential to determine whether nutraceutical interventions can ultimately complement existing therapies, inform novel drug development, and meaningfully contribute to disease-modifying approaches for PD and related synucleinopathies.

## Data Availability

No new data were created or analyzed in this study. The original contributions presented in this study are included in the article.

## Supplementary Materials

The following supporting information can be downloaded at: https://www.mdpi.com/article/10.3390/ijms27031324/s1.

## Abbreviations

The following abbreviations are used in this manuscript:

PD	Parkinson’s disease
α-syn	α-synuclein
DLB	dementia with Lewy bodies
MSA	multiple system atrophy
CSF	cerebrospinal fluid
SNARE	soluble N-ethylmaleimide-sensitive factor attachment protein receptor
NAC	non-amyloid-β component
IDP	intrinsically disordered protein
SMFS	single-molecule force spectroscopy
NMR	nuclear magnetic resonance
cryo-EM	cryo-electron microscopy
LLPS	liquid-liquid phase separation
ROS	reactive oxygen species
ATP	adenosine-5′-triphosphate
EGCG	epigallocatechin gallate
Aβ42	amyloid-β42
BBB	blood-brain barrier
PI3K	phosphatidylinositol 3-kinase
Akt	protein kinase B
CREB	response element-binding protein
BDNF	brain-derived neurotrophic factor
TrkB	tropomyosin receptor kinase B
NF-κB	nuclear factor-kB
AMPK	AMP-activated protein kinase
hIAPP	human islet amyloid polypeptide
B-7-A	brazilin-7-acetate
mTOR	mammalian target of rapamycin
PINK1	PTEN-induced kinase 1
OleA	oleuropein aglycone
PDA-Cur NPs	polydopamine-assembled curcumin nanoparticles
MPTP	1-methyl-4-phenyl-1,2,3,6-tetrahydropyridine
TFEB	transcription factor EB
MOPET	4-hydroxy-3-methoxyphenylethanol
SIRT1	sirtuin 1
Hsp70	Heat shock protein 70
QNEs	quercetin-loaded nanoemulsions
PGC-1α	peroxisome proliferator-activated receptor gamma coactivator 1-alpha
VDAC1	voltage-dependent anion channel 1
nAChRs	nicotinic acetylcholine receptors
SNpc	substantia nigra pars compacta
CNS	central nervous system
FDDNP-PET	a 2-(1-{6-[(2-[F-18]fluoroethyl)(methyl)amino]-2-naphthyl}ethylidene)malononitrile positron emission tomography
CD	Circular Dichroism
SEC	Size Exclusion
AFM	Atomic Force Microscopy
TEM	Transmission Electron Microscopy
IF	Immunofluorescence
ESI–IM–MS	Electrospray ionization-ion mobility mass spectrometry
FRA	Filter Retardation Assay
RT-QuIC	Real-Time Quak-ing-Induced Conversion
FRAP	Fluorescence Recovery After Photobleaching
DIC	Differential Interference Contrast
FTIR	Fourier Transform Infrared
DLS	Dynamic Light Scattering
FRET	Förster Resonance Energy Transfer
HDX-MS	Hydrogen-deuterium exchange mass spectrometry
ITC	Isothermal titration calorimetry
ANS	Anilinonaphthalene-8-sulphonic acid
PCG	Pingchan granules
TCM	Traditional Chinese Medicine
MRI	Magnetic Resonance Imaging
