# Supplementary material for: Interactions Between Nutraceuticals and α-Synuclein Conformational States: Molecular Mechanisms and Neuroprotective Implications in Parkinson’s Disease
Source: Int J Mol Sci. 2026 Jan 28;27(3):1324. doi: 10.3390/ijms27031324 (PMC12898690; doi:10.3390/ijms27031324)
Supplement: Supplementary file 1 [file ijms-27-01324-s001.zip › ijms-4087093-supplementary.pdf]

Supplementary Materials – Table S1: Experimental models and concentration ranges of nutraceuticals used to investigate α-syn aggre-gation and toxicity

Nutraceuticals	Experimental model	Concentration range	References
**BAICALEIN**	SH-SY5Y/HeLa cells	50µM	*Lu, J.-H. et al., 2011*
	Rotenone-induced PD mouse model (C57BL/6)	100 mg/kg i.p.	*Hu, Q. et al., 2016*
	SH-SY5Y/ OLN-93 toxicity<br>Rotenone-induced PD mouse model	16–125 µM<br>100 µM	*Aliakbari, F. et al., 2021*
**BRAZILIN**	PC12 cells	5–100 µM	*Liu, F. et al., 2019*
**CURCUMIN**	SH-SY5Y cells (A53T α-syn-DsRed2)<br>MN9D cells (A53T α-syn)	0.5 -5 µM<br>10^{-7}– 10^{-6} M	*Pandey, N. et al., 2008*
	HEK293 toxicity	50 µM	*Li, J.-F. et al., 2024*
	Differentiated SH-SY5Y cells	~3 µM	*Singh, P.K. et al., 2012*
	SH-SY5Y cells	4 µM	*Wang, M.S. et al., 2010*

	Differentiated SH-SY5Y cells (WT/A53T α-syn)	3–30 μM	*Jiang, T.-F. et al., 2013*
	PC12 rat pheochromocytoma cells (A53T α-syn)	100 nM – 1 μM	*Liu, Z. et al., 2011*
	SH-SY5Y cells (PINK1 knockdown; ± paraquat)	2 μM	*van der Merwe, C. et al., 2017*
**EPIGALLOCATECHIN-3-GALLATE (EGCG)**	HEK-293/PC12 cells	20 μM	*Bieschke, J. et al., 2010*
	PC12 (α-syn overexpression)	1–50 μM	*Zhao, J. et al., 2017*
	Differentiated SH-SY5Y cells	10–70 μM	*Yang, J.E. et al., 2017*
**KAEMPFEROL**	N2a cells (WT/mutant α-syn)	5 μM	*Inden, M. et al., 2021*
	Transgenic Drosophila melanogaster expressing human α-syn	10–40 μM	*Rahul et al., 2020*
**OLEUROPEIN AGLYCONE**	SH-SY5Y cells	1:10 molar ratio	*Palazzi, L. et al., 2018*
	Differentiated SH-SY5Y/ OLN-93 cells	0.025–0.30 mg/mL(extracts)<br>10–200 μM (isolated compounds)	*Mohammad-Beigi, H. et al., 2019*
	SH-SY5Y cells	1:1–1:4	*Palazzi, L. et al., 2020*

**QUERCETIN**	Transgenic Caenorhabditis elegans (CL2659, NL5901) expressing human α-syn-YFP	30–300 μM	*Pretsch, D. et al., 2020*
**RESVERATROL**	SH-SY5Y cells	1–200 μM	*Seyedfatemi, S.S. et al., 2025*
**CAFFEINE**	Saccharomyces cerevisiae	0.1–1 mM	*Kardani, J. et al., 2015*
**NICOTINE**	Saccharomyces cerevisiae (WT, A53T)	0.1–1 mM	*Kardani, J. et al., 2017*
**GINSENOSIDE Rb1**	BE(2)-M17 human neuroblastoma cells	25–100 μM	*Ardah, M.T. et al., 2015*
